# Cost-effectiveness of anti-vascular endothelial growth factor and macular laser treatments for people with centre-involving diabetic macular oedema and central retinal thickness of at least 400 micrometres

**DOI:** 10.1038/s41433-025-04015-6

**Published:** 2025-09-19

**Authors:** Kirsty Luckham, Hannah Tebbs, Clare Dadswell, Ahmed Yosef, Lindsay Claxton, Kate Kelley, Nichole Taske, Philip I. Burgess, Christiana Dinah, Noemi Lois, Syed Mohiuddin

**Affiliations:** 1 Science, Evidence and Analytics Directorate, National Institute for Health and Care Excellence, London, UK; 2 Clinical Evidence, BMJ Technology Assessment Group, London, UK; 3 Centre for Guidelines, National Institute for Health and Care Excellence, London, UK; 4 Department of Eye and Vision Science, University of Liverpool, Liverpool University Hospitals NHS Foundation Trust, Liverpool, UK; 5 Research and Innovation, London North West University Healthcare NHS Trust, London, UK; 6 Wellcome-Wolfson Institute for Experimental Medicine; School of Medicine, Dentistry and Biomedical Sciences, Queen’s University Belfast, Belfast, UK

**Keywords:** Health care economics, Outcomes research

## Abstract

**Background:**

Diabetic macular oedema (DMO) is a common cause of vision loss and blindness. To inform the 2024 UK NICE guideline for treating people with centre-involving DMO (CI-DMO) and central retinal thickness (CRT) of ≥400 µm, the cost-effectiveness of various anti-vascular endothelial growth factor (anti-VEGF) and macular laser treatments was evaluated.

**Methods:**

A de novo Markov model evaluated the lifetime costs and quality-adjusted life-years (QALYs) of various anti-VEGFs (aflibercept, bevacizumab, brolucizumab, faricimab, ranibizumab and ranibizumab biosimilar), macular lasers (standard threshold laser and subthreshold micropulse laser), and some treatment combinations from the perspective of the UK NHS. The model included eight health states defined by best-corrected visual acuity ranging between >85 and ≤25 letters. The model’s inputs were derived from published literature, while an original network meta-analysis of several clinical trials informed visual outcomes.

**Results:**

All anti-VEGFs demonstrated greater clinical effectiveness and produced more QALYs (ranging from 9.211 to 9.271) than both types of macular lasers (8.928 and 8.944), but lasers were the most cost-effective due to their substantially lower costs. Using confidential price discounts, ranibizumab biosimilar (Ongavia) and brolucizumab had ICERs below £20,000 per QALY, while aflibercept, ranibizumab (Lucentis) and faricimab had ICERs below £25,000 per QALY, compared to no treatment. Bevacizumab was the most cost-effective anti-VEGF treatment due to its significantly lower cost.

**Conclusions:**

Given their clinical and cost-effectiveness at confidential prices, NICE recommends offering a licensed cost-effective anti-VEGF as first-line treatment for people with CI-DMO and CRT ≥ 400 µm. The use of bevacizumab for this population is not licensed in the UK and would be considered off-label.

## Introduction

Diabetic macular oedema (DMO) is a leading cause of vision loss and blindness worldwide [1]. Approximately 7.5% of people living with diabetes develop DMO [2], which can profoundly affect their quality of life (QoL) and ability to work [3, 4]. People with DMO use significantly more healthcare resources than those with diabetes who do not have retinal complications [5]. DMO can be classified as non-centre-involving DMO and centre-involving DMO (CI-DMO); the latter defined by the presence of fluid in the central 1 mm of the retina—referred to as the central subfield thickness or central retinal thickness (CRT). CI-DMO can be mild (CRT < 400 µm) or severe ( ≥ 400 µm) [6].

For people with CI-DMO and CRT < 400 µm, macular laser treatment is considered a highly cost-effective option [6]. However, in the study by Mansouri et al. [7], all eyes with an initial CRT≥400μm did not respond to laser treatment and subsequently required rescue therapy with anti-vascular endothelial growth factor (anti-VEGF) injections. During the past years, several intravitreal anti-VEGF and steroid therapies were successfully introduced for the management of DMO. To manage people with CI-DMO and CRT ≥ 400 µm, the National Institute for Health and Care Excellence (NICE) in the UK supports the use of aflibercept [8], brolucizumab [9], faricimab [10] and ranibizumab [11]. Although anti-VEGFs can be administered by various healthcare professionals, they are generally expensive, require prolonged treatment courses, and raise significant concerns regarding cost-effectiveness [12, 13].

While randomised controlled trials (RCTs) have demonstrated the clinical effectiveness of anti-VEGFs in treating visual impairment due to CI-DMO and CRT ≥ 400 µm, no RCT has yet compared all available anti-VEGFs, nor has any assessed their cost-effectiveness in a single analysis. There is a compelling public health argument for selecting safe treatments that enhance vision and improve health outcomes, while ensuring an acceptable balance between health benefits and costs. Informing the 2024 UK NICE guideline [14], this study aimed to evaluate the cost-effectiveness of various anti-VEGFs, macular lasers, and some treatment combinations in a single analysis for treating people with CI-DMO and CRT ≥ 400 µm within a UK NHS setting.

## Methods

### Model overview and structure

A cohort Markov model with a 3-monthly cycle length and a half-cycle correction was developed in Microsoft® Excel® to evaluate the lifetime costs and quality-adjusted life-years (QALYs) of eleven treatment strategies for people with CI-DMO and CRT ≥ 400 µm, from the perspective of UK NHS. The strategies evaluated included: aflibercept (Eyela) 2 mg, bevacizumab (Avastin) (off-label) 1.25 mg, brolucizumab (Beovu) 6 mg, faricimab (Vabysmo) 6 mg, ranibizumab (Lucentis) 500 µg, ranibizumab biosimilar (Ongavia) 500 µg, standard threshold laser, subthreshold micropulse laser, bevacizumab plus standard threshold laser, ranibizumab plus standard threshold laser, and no treatment. It was assumed that the ranibizumab biosimilar (Ongavia) would have the same efficacy, safety and resource use as ranibizumab (Lucentis). All costs and QALYs were discounted at an annual rate of 3.5% [15].

Macular laser treatment, delivered via either standard threshold or subthreshold micropulse laser, was included in the analysis to allow comparison with other treatment options, as most RCTs comparing anti-VEGF therapy with macular laser involved participants with CRT well over 400 µm [16]. Although laser treatments are predominantly used in people with CI-DMO and CRT < 400 µm, including them as a comparator for the CRT ≥ 400 µm subgroup allowed for a more complete network and enabled the inclusion of additional studies. Intravitreal steroids, such as dexamethasone [17] and fluocinolone [18], were excluded, as steroids are primarily used as second-line treatments and are only considered first-line treatments for people who are not suitable for other first-line treatments. This study focused solely on first-line treatments.

**Fig. 1:**
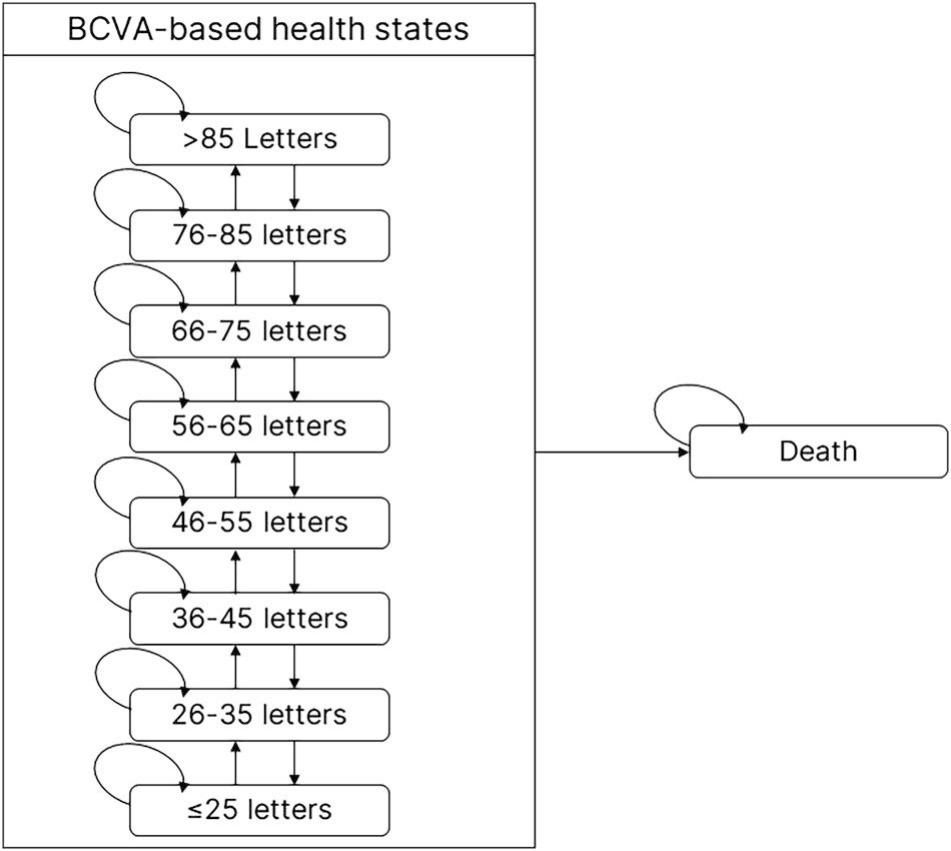
Schematic diagram of the Markov model showing possible health state transitions.

As shown in Fig. 1, the Markov model consisted of nine health states, including eight levels of best-corrected visual acuity (BCVA) ranging between >85 and ≤25 letters and a death state. Based on clinical input, the model allowed people to transition by up to one BCVA state in each 3-monthly cycle (i.e., a BCVA change of 5 to 15 letters; a 10-letter range), reflecting the average eye rather than using the midpoint. The rationale for adopting a BCVA-based model (even though it may not fully capture all outcomes related to disease progression) was to incorporate the primary outcome data from RCTs and to validate the results against previously published cost-effectiveness analyses.

### Baseline data

The cohort in the model began at the age of 63 years, with 58% being male [19]. The model assumed an initial distribution of people across BCVA-based health states at baseline (Table 1). In line with previous BCVA-based models [19–22], the model structure was based on one eye only. However, as in NICE TA799 [10] and NICE TA820 [9], the costs for treating the fellow eye were also included, assuming that 22% of people received treatment in both eyes at baseline [22]. Of those treated in one eye, 67.2% received treatment in their worst seeing eye (WSE) and 32.8% in their best seeing eye (BSE) [19]. The probability of developing DMO in the fellow eye was 5.4% per 3-monthly cycle [21]. The parameters used in the model are shown in Table 1.

**Table 1:** Various parameters used in the model.

Parameter	Point estimate	Probabilistic analysis		Sources
		Distribution	Parameters	
**Baseline starting distribution of BCVA in each health state for treated eye**				
BCVA: >85	0%	Dirichlet	Not applicable	Régnier et al. [22]
BCVA: 76-85	11%			
BCVA: 66-75	39%			
BCVA: 56-65	27%			
BCVA: 46-55	15%			
BCVA: 36-45	8%			
BCVA: 26-35	0%			
BCVA: ≤25	0%			
**Fellow eye involvement**				
Probability of DMO in the fellow eye	5.4%	Beta	α = 7.900, β = 138.397	Pochopien et al. [21]
Patients treated in both eyes	22.0%	Beta	α = 75.900, β = 269.100	Régnier et al. [22]
Patients treated in worst seeing eye	67.2%	Dirichlet	Not applicable	Mitchell et al. [19]
Patients treated in best seeing eye	32.8%			
**Natural history**				
Probability of gaining one health state	3.5%	Dirichlet	Not applicable	Mitchell et al. [19]
Probability of losing one health state	4.5%			
Probability of staying in health state	92.0%			
**Mortality hazard ratio (HR) for diabetes**				
Mortality HR diabetes	1.950	Lognormal	μ = 0.668, σ = 0.090	Preis et al. [28]
**Treatment effect at 12 months (mean difference, LogMAR)**				
Aflibercept (vs. no treatment)	-0.286	Normal	μ = -0.286, σ = 0.060	Network meta-analysis
Bevacizumab (vs. no treatment)	-0.220		μ = -0.220, σ = 0.059	
Brolucizumab (vs. no treatment)	-0.285		μ = -0.285, σ = 0.067	
Faricimab (vs. no treatment)	-0.303		μ = -0.303, σ = 0.066	
Ranibizumab^a^ (vs. no treatment)	-0.234		μ = -0.234, σ = 0.056	
Standard threshold laser (vs. no treatment)	-0.087		μ = -0.087, σ = 0.059	
Subthreshold micropulse laser (vs. no treatment)	-0.087		μ = -0.087, σ = 0.059	
Bevacizumab plus standard laser (vs. no treatment)	-0.222		μ = -0.222, σ = 0.060	
Ranibizumab^a^ plus standard laser (vs. no treatment)	-0.222		μ = -0.222, σ = 0.060	

^a^ Ranibizumab biosimilar (Ongavia) was assumed to have the same efficacy as ranibizumab (Lucentis).

*BCVA* best-corrected visual acuity, *DMO* diabetic macular oedema, *LogMAR* logarithm of the minimum angle of resolution.

### Natural history and treatment efficacy

The natural history of DMO was informed by Mitchell et al. [19], who used data from the Wisconsin epidemiologic study of diabetic retinopathy (WESDR) to create a transition matrix for the natural progression of DMO. This was recalibrated with data from the RESTORE trial, yielding 3-monthly probabilities of 3.5% for moving up and 4.5% for moving down one health state (Table 1). The natural history was applied to the no treatment arm and other interventions when treatment efficacy was assumed to have ceased in scenario analyses.

Mean treatment effects with 95% confidence intervals, measured as changes in BCVA at 12 months, were derived from an original network meta-analysis (NMA), described elsewhere [23], which compared each treatment to no treatment. The NMA included 32 RCTs with a total sample size of 7,721 participants; a forest plot is shown in Fig. S1 in Supplementary material. Although several RCTs enroled participants with CRT both above and below 400 µm, only those with CRT ≥ 400 µm were included in the NMA. Using these data, 3-monthly probabilities of transitioning between different health states were calculated (Table S1; Supplementary material), assuming that changes in BCVA follow a normal distribution [24]. The efficacy of the subthreshold laser was assumed to be equivalent to that of the standard threshold laser given their similar performance in the DIAMONDS clinical trial [25]. In the absence of data, it was assumed that the efficacy and resource use of bevacizumab plus standard threshold laser would be equal to those of ranibizumab plus standard threshold laser.

In the base-case analysis, the model assumed that the treatment effects observed in the NMA would continue throughout the model’s lifetime. However, scenario analyses assessed the impact of including natural history after 5, 10 and 20 years (i.e., treatment effects ceased at 5, 10 and 20 years, after which natural disease progression resumed).

### Treatment discontinuation

The model assumed that everyone would continue treatment during the first year to assess their response (clinical consensus). The published treatment-specific discontinuation rates were applied between years 1 and 3 (Table S2; Supplementary material). From years 3 to 5, the model assumed that 75% of people would continue treatment [26], and from year 5 onward, 50% would continue treatment [10]. The treatment-specific discontinuation rates were applied alongside the fixed treatment discontinuation starting from year 3 onward.

### Adverse events (AEs)

AEs related to each treatment were included in the model. In the absence of data, it was assumed that the frequency of AEs for subthreshold micropulse laser would be the same as that for standard threshold laser, ranibizumab plus standard threshold laser would be the same as for ranibizumab, and bevacizumab plus standard threshold laser would be the same as for bevacizumab. The proportion of each AE for each treatment strategy is shown in Table S3 (Supplementary material), while the associated cost of each AE is provided in Table S4 (Supplementary material).

### Mortality

Mortality was modelled using age- and gender-specific national life tables for England and Wales (2018–2020) [27]. It was anticipated that the main mortality risk associated with poor vision would be reflected in the diabetes population rather than in the diabetic retinopathy population. Therefore, following the approach in NICE TA346 [8], a hazard ratio of 1.95 [28] was applied to account for the increased mortality risk associated with diabetes relative to the general population.

### Resource use and costs

The costs associated with treatment (list prices), administration, monitoring and low vision (BCVA ≤ 35 letters) are shown in Table 2. The cost year 2019-20 was chosen to represent standard care in the NHS and to avoid any cost outliers caused by the COVID-19 outbreak.

**Table 2:** Cost and utility parameters used in the model.

Cost/Utility	Point estimate	Probabilistic analysis^a^		Sources/Notes
		Distribution	Parameters	
**Treatment cost (list price)**				
Aflibercept 4.0 mg/0.1 ml	£816.00	Gamma	μ = 96.036, σ = 8.497	BNF 28/03/2023 bnf.nice.org.uk
Brolucizumab 19.8 mg/0.165 ml	£816.00		μ = 96.036, σ = 8.497	
Faricimab 28.8 mg/0.24 ml	£857.00		μ = 96.036, σ = 8.924	
Ranibizumab (Lucentis) 2.3 mg/0.23 ml	£551.00		μ = 96.036, σ = 5.737	
Ranibizumab biosimilar (Ongavia) 2.3 mg/0.23 ml	£523.45^b^		μ = 96.036, σ = 5.451	
Bevacizumab 1.25 mg	£50.00^c^		μ = 96.036, σ = 0.521	NICE TA824 [17]
Standard threshold laser	£41.16		μ = 96.036, σ = 0.429	Lois et al. [25]
Subthreshold micropulse laser	£47.11		μ = 96.036, σ = 0.491	Lois et al. [25]
**Administration cost for anti-VEGFs**				
Optical coherence tomography (applied to 100% of visits)	£101.804	Gamma	μ = 96.036, σ = 1.060	NHS reference costs 2019-20. Consultant led non-admitted face-to-face attendance, follow-up. Code 130 (ophthalmology). Assumption used in NICE TA294 [41].
Administration visit – outpatient (applied to 95% of visits)	£129.616		μ = 96.036, σ = 1.350	NHS reference costs 2019-20. Outpatient procedure. BZ87A minor vitreous retinal procedures. Assumption used in NICE TA294 [41].
Administration visit - day case (applied to 5% of visits)	£660.838		μ = 96.036, σ = 6.881	NHS reference costs 2019-20. Day case procedure. BZ87A minor vitreous retinal procedures. Assumption used in NICE TA294 [41].
Anti-VEGF administration per visit	£257.981	Not applicable	Not applicable	Calculation based on the above inputs.
**Monitoring cost**				
Monitoring visit during treatment	£101.804	Gamma	μ = 96.036, σ = 1.060	NHS reference costs 2019-20. Consultant led non-admitted face-to-face attendance, follow-up. Code 130 (ophthalmology). Assumption used in NICE TA294 [41].
Monitoring visit post treatment	£38.344		μ = 96.036, σ = 0.399	£32 (2012-13) from Scanlon et al. [42] was inflated to 2019-20 prices.
**Low vision cost per 3-monthly cycle**				
Healthcare costs for low vision	£421.609	Gamma	μ = 25.003, σ = 16.862	Régnier et al. [22] The yearly total cost of visual impairment (BCVA ≤ 35) is £17,326, minus the cost of residential care (£15,327), community care (£600) and low vision rehabilitation (£47), in alignment with the NHS perspective. The costs (from 2010-11) were inflated to 2019-20 prices and then adjusted to a 3-monthly cycle length.
**Utility for best seeing eye (treated eye)**				
BCVA: >85	0.860	Beta	α = 88.711, β = 14.441	Czoski-Murray et al. [29] The authors used contact lenses with varying central opacity to simulate different stages of AMD, with health states valued by members of the public. This approach might have led to an underestimation of utility in more severe vision loss and an overestimation of treatment effects. Nonetheless, it was considered the most appropriate source due to its widespread use in previous NICE TAs for DMO [8, 17, 18], its alignment with the NICE reference case, and its inclusion in the NICE AMD guideline [43].
BCVA: 76-85	0.860		α = 527.426, β = 85.860	
BCVA: 66-75	0.813		α = 857.529, β = 197.242	
BCVA: 56-65	0.802		α = 648.965, β = 160.218	
BCVA: 46-55	0.770		α = 420.116, β = 125.489	
BCVA: 36-45	0.760		α = 189.396, β = 59.809	
BCVA: 26-35	0.681		α = 51.985, β = 24.351	
BCVA: ≤25	0.547		α = 19.128, β = 15.841	
**Utility for worst seeing eye (treated eye)**				
BCVA: >85	0.860	Beta	α = 88.711, β = 14.441	The utility value for BCVA > 85 in the worst seeing eye was set equal to the value for BCVA > 85 in the best seeing eye, as reported by Czoski-Murray et al. [29]. Similar to the approach used by Régnier et al. [22], a utility decrement of 0.1 was assumed between the best (BCVA > 85) and worst (BCVA ≤ 25) health states, with a linear decline assumed for calculating the utility values of the other states.
BCVA: 76-85	0.860		α = 527.426, β = 85.860	
BCVA: 66-75	0.843		α = 772.928, β = 143.587	
BCVA: 56-65	0.827		α = 603.521, β = 126.545	
BCVA: 46-55	0.810		α = 383.940, β = 90.060	
BCVA: 36-45	0.793		α = 177.631, β = 46.274	
BCVA: 26-35	0.777		α = 47.182, β = 13.567	
BCVA: ≤25	0.760		α = 19.363, β = 6.114	

^a^ Varied by ±20% when relevant data were not available.

^b^ Assuming the same efficacy, safety and resource use as ranibizumab (Lucentis).

^c^ This cost of £50 per 1.25 mg dose was used, as it is approximately the price clinics would pay and is also aligned with previous TA.

AMD (age-related macular degeneration); BCVA (best-corrected visual acuity); BNF (British National Formulary); HRGs (healthcare resource groups); NHS (National Health Service); NICE (National Institute for Health and Care excellence); TA (technology appraisal).

Aflibercept, brolucizumab, faricimab, ranibizumab (Lucentis) and ranibizumab biosimilar (Ongavia) have confidential patient access scheme prices to NICE; these prices cannot be disclosed due to confidentiality agreements. A weighted cost of £257.98 was applied for administering an anti-VEGF (Table 2), while the cost of laser administration was assumed to be included within the overall laser treatment cost. It was also assumed that treatment would be administered during the same visit as monitoring, and that both eyes would receive the same treatment during that visit for those who require it. Treatment monitoring costs covered the cost of an optical coherence tomography scan, as well as the cost of a post-treatment monitoring visit (Table 2). The number of monitoring visits for each treatment and the annual number of anti-VEGF injections are shown in Tables S5 and S6 (Supplementary Material), respectively. The number of laser treatments is shown in Table S7 (Supplementary material), with the assumption that all combination treatment options involved the same number of laser treatments. The total cost of AEs for each treatment was calculated by multiplying the proportion of people experiencing an AE (Table S3; Supplementary material) by the cost of that AE (Table S4; Supplementary material).

Some people may switch to a different treatment after discontinuing the previous one due to lack of response. However, there is significant variability in how subsequent treatments are reported in the literature, and no data were available on the duration of these treatments. The model allowed some people to receive a subsequent treatment (Table S8; Supplementary material), but this was applied to costs for only two years to prevent overestimating the cost of first-line treatment by including the additional cost of the subsequent treatment, for which evidence is limited. The total cost of subsequent treatment expected for each first-line treatment strategy is shown in Table S9 (Supplementary material). The costs were calculated based on the weightings shown in Table S8 (Supplementary material), including the costs of treatment itself, administration and treatment-specific AEs.

### Health state utility values

Utility values were obtained from a review of QoL literature and are summarised in Table 2. Although several studies estimated utility values based on BCVA, the utility values for BSE from Czoski-Murray et al. [29] were considered the most relevant and were also used in NICE technology appraisals (TAs) for DMO [8, 17, 18]. Consistent with Haig et al. [20], the utility values for BSE were applied to people receiving treatment in both eyes, as BSE is identified as the primary factor influencing overall QoL and functioning [19, 30]. QALYs were calculated by multiplying the time spent in each BCVA-based health state by its corresponding utility value, with adjustments made for utility losses due to treatment-related adverse events. In addition to the health state utility values, the model also included utility losses associated with presumed AEs (Table S10; Supplementary material).

### Deterministic and probabilistic sensitivity analyses

The following deterministic scenario analyses were performed:

- Treatment efficacy ceased and natural history starts from 20 years.
- Treatment efficacy ceased and natural history starts from 10 years.
- Treatment efficacy ceased and natural history starts from 5 years.
- 25% of people continue treatment after 5 years.
- 75% of people continue treatment after 5 years.
- Treatment occurs in a separate visit from monitoring.
- Monitoring and treatment assumption (minimum).
- Monitoring and treatment assumption (maximum).

The probabilistic sensitivity analysis was performed to assess the uncertainty in the true values of the input parameters. Probability distributions were assigned to the input parameters, with the distribution type chosen based on the properties of the data. Where possible, dispersion data from the original source were used to parameterise each distribution. In the absence of such data, expert judgement was used to apply reasonable ranges.

## Results

The probabilistic lifetime cost-effectiveness results, based on list prices, are shown in Table 3. All anti-VEGF treatments generated more QALYs (ranging from 9.211 to 9.271) than either type of macular laser (8.928 and 8.944), but the lasers were substantially less expensive (£4,458 and £4,919) compared to the anti-VEGF treatments (ranging from £9,308 to £34,522). When evaluating cost-effectiveness relative to no treatment using the incremental cost-effectiveness ratio (ICER), lasers were found to be the most cost-effective treatment option due to their significantly lower costs. Bevacizumab (off-label) became the most cost-effective anti-VEGF treatment due to its lower cost compared to all other anti-VEGFs, despite producing fewer QALYs than the others. However, with confidential price discounts, ranibizumab biosimilar (Ongavia) and brolucizumab had ICERs below NICE’s lower threshold of £20,000 per QALY, while aflibercept, ranibizumab (Lucentis) and faricimab had ICERs below £25,000 per QALY, compared to no treatment. The ICER was calculated by dividing the difference in costs by the difference in QALYs, indicating that the high ICERs for anti-VEGF therapies were primarily driven by their higher incremental costs. Each strategy was compared to no treatment rather than using a full incremental analysis, as laser treatment is not clinically recommended for this population.

**Table 3:** Probabilistic cost-effectiveness results compared to no treatment using list prices.

Strategy	Total cost per person	Total QALYs^a^ per person	ICER	NMB^b^ [95% CI] at £20,000 per QALY	Ranked^c^ by NMB	Ranked^d^ by NMB
No treatment	£3,822	8.503	-	£166,238 [£152,957 – £180,234]	5	8
Subthreshold micropulse laser	£4,458	8.944	£1,442	£174,414 [£160,952 – £187,227]	2	1
Standard threshold laser	£4,919	8.928	£2,579	£173,646 [£159,605 – £187,244]	3	3
Bevacizumab	£9,308	9.211	£7,746	£174,916 [£161,429 – £187,533]	1	2
Bevacizumab plus standard threshold laser	£11,325	9.211	£10,593	£172,899 [£159,168 – £186,269]	4	4
Ranibizumab biosimilar (Ongavia)	£23,252	9.225	£26,276	£161,251 [£147,381 – £174,920]	6	5
Ranibizumab (Lucentis)	£24,039	9.224	£28,054	£160,434 [£146,828 – £174,059]	8	10
Ranibizumab (Ongavia) plus standard threshold laser	£24,164	9.210	£28,073	£160,043 [£146,307 – £173,534]	9	7
Brolucizumab	£24,348	9.268	£26,824	£161,016 [£147,669 – £173,755]	7	6
Ranibizumab (Lucentis) plus standard threshold laser	£24,904	9.209	£29,878	£159,268 [£145,571 – £172,882]	10	12
Faricimab	£33,979	9.271	£39,250	£151,448 [£137,073 – £164,968]	11	11
Aflibercept	£34,522	9.267	£40,196	£150,813 [£136,809 – £164,845]	12	9

^a^ QALYs were calculated by multiplying the time spent in a health state by the corresponding utility value for that state, while adjusting for utility losses due to adverse events associated with the treatment.

^b^ NMB was calculated using the formula [(QALYs x £20,000) – Cost]; the strategy having the highest NMB considered the most cost-effective.

^c^ The ranking is based on the list prices.

^d^ The ranking is based on the confidential patient access scheme prices available to NICE for aflibercept, brolucizumab, faricimab, ranibizumab (Lucentis) and ranibizumab biosimilar (Ongavia). These prices and the results using them cannot be disclosed due to confidentiality agreements

*ICER* incremental cost-effectiveness ratio, NMB net monetary benefit, *QALYs* quality-adjusted life years.

Based on net monetary benefit (NMB) estimates at the £20,000 per QALY gained threshold, bevacizumab had the highest NMB (£174,916), followed by subthreshold micropulse laser with the second-highest NMB (£174,414) and standard threshold laser with the third-highest NMB (£173,646) in the base-case analysis using list prices. The results involving confidential prices cannot be disclosed due to confidentiality agreements. However, the NMB rankings of the interventions, calculated using both list and confidential prices, are shown in Table 3.

Table 4 shows a summary of the probabilistic cost-effectiveness results from various scenario analyses, including the base-case. In all the scenarios explored, bevacizumab, subthreshold micropulse laser and standard threshold laser consistently ranked among the top three interventions. While the use of confidential prices did not allow other interventions to enter the top three across all scenarios, it did cause some changes in the rankings compared to using list prices. The model results were robust to most parameters explored in the scenario analyses. However, all anti-VEGF options were somewhat impacted by changes in assumptions about the frequency of treatment and monitoring visits, as these factors affected the associated costs.

**Table 4:** Summary of probabilistic cost-effectiveness results of scenario analyses using list prices.

Scenario	Treatment ranking best NMB^a^ [95% CI]	Treatment ranking 2nd best NMB^a^ [95% CI]	Treatment ranking 3rd best NMB^a^ [95% CI]
Base-case	Subthreshold laser £174,414 [£160,952 – £187,227]	Bevacizumab £174,916 [£161,429 – £187,533]	Standard laser £173,646 [£159,605 – £187,244]
Treatment efficacy ceased and natural history starts from 20 years	Bevacizumab £174,859 [£161,619 – £189,552]	Subthreshold laser £174,238 [£160,078 – £189,540]	Standard laser £173,961 [£160,318 – £188,203]
Treatment efficacy ceased and natural history starts from 10 years	Subthreshold laser £172,451 [£159,238 – £185,078]	Standard laser £171,984 [£158,276 – £185,321]	Bevacizumab £171,813 [£159,075 – £184,042]
Treatment efficacy ceased and natural history starts from 5 years	Subthreshold laser £169,896 [£156,990 – £182,867]	Standard laser £169,513 [£156,438 – £182,680]	Bevacizumab £167,528 [£154,558 – £180,516]
25% of people continue treatment after 5 years	Bevacizumab £175,816 [£162,789 – £188,051]	Bevacizumab + Standard laser £174,313 [£161,091 – £186,674]	Subthreshold laser £174,172 [£160,535 – £188,263]
75% of people continue treatment after 5 years	Bevacizumab £174,689 [£161,288 – £187,777]	Subthreshold laser £174,617 [£159,793 – £187,239]	Standard laser £174,151 [£160,186 – £188,096]
Treatment occurs in a separate visit from monitoring	Subthreshold laser £173,774 [£160,234 – £187,907]	Standard laser £173,553 [£159,898 – £187,748]	Bevacizumab £172,929 [£159,819 – £186,044]
Monitoring and treatment assumption (minimum)	Bevacizumab £177,514 [£164,053 – £191,212]	Subthreshold laser £174,987 [£160,622 – £189,146]	Standard laser £174,647 [£159,315 – £189,187]
Monitoring and treatment assumption (maximum)	Subthreshold laser £172,953 [£158,548 – £187,519]	Standard laser £172,497 [£158,394 – £186,878]	Bevacizumab £171,971 [£159,158 – £185,572]

^a^ NMB was calculated using the formula [(QALYs x £20,000) – Cost]; the strategy having the highest NMB considered the most cost-effective.

*DMO* diabetic macular oedema, *NMB* net monetary benefit, *QALYs* quality-adjusted life years.

## Discussion

The cost-effectiveness of various anti-VEGFs and macular lasers was evaluated to inform the 2024 NICE guideline update for treating people with CI-DMO and CRT ≥ 400 µm. Although anti-VEGFs were more clinically effective (Table S1; Supplementary material) and produced more QALYs (Table 3) than either type of laser, both lasers emerged as the most cost-effective treatment option due to their very low costs. However, with confidential price discounts, all anti-VEGFs were cost-effective compared to no treatment, with ICERs either below £20,000 or £25,000 per QALY. NICE considers interventions costing between £20,000 and £30,000 per additional QALY gained to represent good value for money for the NHS. Bevacizumab was identified as the most cost-effective anti-VEGF treatment; however, its use in the UK would be considered off-label as it is not covered by its marketing authorisation.

A key strength of this study is that the mean difference in BCVA used to inform the model was derived from data across multiple RCTs included in the NMA. However, several of these RCTs reported only aggregated outcomes for the overall study population, without stratifying results by CRT subgroups. For this analysis, RCTs were categorised based on whether their mean baseline CRT was above or below 400 µm. A limitation of this approach is that individuals with a CRT below 400 µm may have been included in the ‘above 400 µm’ subgroup if the RCT’s mean CRT exceeded that threshold (and vice versa). Fewer RCTs assessed treatment strategies for individuals with CRT < 400 µm, resulting in insufficient data to perform a comparable analysis for this subgroup.

Another key strength of this study is that the model compared all relevant treatments in a single analysis, an aspect that had not been addressed in the published literature previously. All parameters used in the model were informed by RCTs, published literature or assumptions, and were verified by clinical experts. The model included people treated in their WSE, BSE or both eyes, along with any subsequent treatments and treatment-related AEs. The model was developed in close collaboration with clinical experts, with meetings held at various stages of the development process to review the model structure and input parameters for clinical relevance.

The model’s base case assumed that the treatment effects observed in the NMA would persist throughout the model’s lifetime, as the two primary reasons for discontinuation were reported to be death and early discharge due to stable disease [31]. This assumption may have led to an overestimation of the treatment effect, as a small number of individuals were reported to have discontinued due to treatment being considered futile, and treatment effect typically declines when assessments or retreatments are delayed [32, 33]. However, assuming that treatment effect would cease immediately upon discontinuation is problematic, as the risk of vision loss in those who discontinue would likely develop gradually over time, and people may return to the same treatment if signs of vision loss appear. Scenario analyses assessed the impact of treatment effects being ceased after 5, 10 and 20 years.

Although the analysis benefited from incorporating AEs, there is inconsistency in how AEs are reported in the literature. Since the overall costs and disutilities associated with AEs included in the model were applied to a very small proportion of people, it is unlikely to impact the conclusions drawn. Mitchell et al. [19] excluded AEs from their analysis based on the assumption that they had a negligible impact on cost-effectiveness. The model allowed people to switch between standard threshold laser and some anti-VEGF treatments, but no switches within the therapeutic class were permitted. However, limited or poorly reported data were available to determine the frequency and distributions of these subsequent treatments, and no data were available on the distribution of treatments for those switching from combination regimens. The duration of subsequent treatment was restricted to two years to avoid overestimating the costs associated with it, given the uncertainty surrounding the true distribution and duration of subsequent treatments. As a result, it is likely that the costs of subsequent treatment were underestimated. However, the analysis benefitted from being more representative of clinical practice by incorporating subsequent treatment at all. The model did not include a separate risk for proliferative diabetic retinopathy (PDR), as most clinical studies report data on PDR and DMO separately. However, the impact of PDR was expected to be captured within the BCVA-based transition probabilities. This approach is consistent with previously published cost-effectiveness models [19, 20, 22].

Patient time and productivity losses were excluded from the analysis to align with the NICE reference case [15], which adopts the NHS perspective in the UK. Although these costs can place a significant burden on patients, aggregating individual patient preferences into a societal preference remains both a theoretical and practical challenge [12]. Future clinical trials may consider collecting these cost data to help address this issue. However, direct healthcare-related costs associated with low vision were considered for people with BCVA ≤ 35 ETDRS letters, including the costs of residential care, community care and low vision rehabilitation.

Given the nature of DMO, both eyes may be affected over time and might require treatment. Since most clinical studies focus on the study eye, the data needed to construct a two-eye model could not be obtained. Consistent with prior research, the BCVA-based health states were based on the study eye, though treatment costs for the fellow eye were included in the model. While this may not fully capture the disease profile, the Markov model structure is widely used in the literature. A discrete event simulation model would have been better suited to capture heterogeneity in disease profile [34, 35]; however, the lack of publicly available patient-level data prevented its development. Instead, a simple and flexible Markov model structure was adopted to enhance transparency and accessibility, ensuring the model remained uncomplicated and enabling a broad range of stakeholders to critically appraise it.

The current analysis aligns with the findings of Lois et al. [25], who found that both standard and subthreshold macular laser were equally effective in people with CI-DMO and CRT < 400 µm, and that both were considered cost-effective. The current analysis also aligns with Sharma et al. [36], who found that laser treatment alone was cost-effective compared to no treatment. Consistent with Mitchell et al. [19], the current analysis found that combined ranibizumab and laser treatment was less cost-effective than ranibizumab alone, primarily because ranibizumab alone produced a higher QALY gain than the combination treatment.

Hutton et al. [37] found that aflibercept was less cost-effective than bevacizumab, which is in agreement with the current analysis. Brown et al. [38] found that ranibizumab was cost-effective over sham, with an ICER of $4,587 per QALY. In contrast, the current analysis found that ranibizumab (Lucentis) was cost-effective over no treatment only when the ICER was below £25,000 per QALY, but the ranibizumab biosimilar (Ongavia) was cost-effective with an ICER below £20,000 per QALY. Holekamp et al. [39] found that aflibercept was less cost-effective than ranibizumab. While the current analysis, based on list prices, aligns with this, the same conclusion does not hold when confidential prices are used—unless the ranibizumab biosimilar replaces ranibizumab. This difference is likely due to variations in the drug prices used in the analysis. Régnier et al. [22] found that ranibizumab was dominant (less expensive and more effective) over aflibercept. In contrast, the current analysis found aflibercept to be more effective. This discrepancy may be due to variations in mean BCVA gain, as the Régnier et al. [22] analysis was based solely on the RESTORE trial, while the current analysis included data from several RCTs. Similar to Stein et al. [40], the current analysis found that bevacizumab was the most cost-effective option among the anti-VEGF treatments examined, primarily due to its substantially lower drug cost.

All anti-VEGFs produced higher QALYs than macular lasers. Off-label bevacizumab (QALYs: 9.204) emerged as a highly cost-effective option, yielding nearly as many QALYs as ranibizumab (QALYs: 9.218), due to its lower cost than all other anti-VEGFs. Faricimab (9.267), brolucizumab (9.262) and aflibercept (9.258) achieved the highest QALYs among all interventions. When confidential prices were factored in, all anti-VEGFs had ICERs either below £20,000 or £25,000 per QALY compared to no treatment. Recommendations for the use of anti-VEGFs referenced the relevant NICE TAs for aflibercept [8], brolucizumab [9], faricimab [10] and ranibizumab [11] in people with CI-DMO and CRT ≥ 400 µm, aligning with the current analysis and ensuring consistency with the evidence base. Although anti-VEGFs require more clinic visits and frequent retreatment compared to either type of macular laser, the benefits of improved vision and the relatively low risk of AEs are expected to outweigh this factor. The introduction of additional biosimilar agents, as patents expire, may further enhance the cost-effectiveness of newer anti-VEGF treatments. Notably, for people with CI-DMO and CRT≥400μm, laser treatment was not as clinically effective as anti-VEGFs and ultimately required rescue treatment with anti-VEGF injections [7].

This study incorporated a substantial body of the best available evidence and offers value to both clinicians and health service planners. However, there is still a need for discussions between clinicians and patients regarding the advantages and disadvantages of the available anti-VEGF treatments. A clear understanding of the available options, their potential benefits, and associated trade-offs can help guide and enrich such discussions within the context of a broad care plan. Since the dosage and timing guidance vary among anti-VEGFs, the information outlined in the summary of product characteristics should be followed by clinicians.

## Conclusions

Macular laser treatment (either standard threshold laser or subthreshold micropulse laser) emerged as the most cost-effective option due to its substantially lower costs. However, all anti-VEGF treatments showed greater clinical effectiveness and produced more QALYs than either type of macular laser in people with CI-DMO and CRT ≥ 400 µm. Furthermore, with confidential price discounts, ranibizumab biosimilar (Ongavia) and brolucizumab had ICERs below NICE’s lower threshold of £20,000 per QALY, while aflibercept, ranibizumab (Lucentis) and faricimab had ICERs below £25,000 per QALY (middle of NICE’s acceptable lower and upper cost-effectiveness thresholds), compared to no treatment. Given their clinical and cost-effectiveness at confidential prices, the 2024 UK NICE guideline recommends offering a licensed cost-effective anti-VEGF as the first-line treatment for people with CI-DMO and CRT ≥ 400 µm. Among the licensed anti-VEGF treatments analysed, ranibizumab biosimilar (Ongavia) and brolucizumab were the two most cost-effective options. Although bevacizumab was the most cost-effective anti-VEGF treatment, its use in the UK would be considered off-label as it is not covered by its marketing authorisation for ophthalmic conditions.

## Summary

### What was known before

- Multiple anti-VEGF agents are approved by NICE technology appraisals for the treatment of people with CI-DMO and CRT of at least 400 micrometres.

### What this study adds

- For people with CI-DMO and CRT of at least 400 micrometres, macular laser (standard or subthreshold) emerged as the most cost-effective treatment option due to its significantly lower costs, although all anti-VEGF treatments produced higher QALYs than either type of macular laser.
- Anti-VEGFs were found to be cost-effective compared to no treatment for people with CI-DMO and CRT of at least 400 micrometres, in reducing the risk of visual impairment, when confidential drug prices were applied.

## Supplementary information

Supplementary material
