# Supplementary material for: Cost-effectiveness of anti-vascular endothelial growth factor and macular laser treatments for people with centre-involving diabetic macular oedema and central retinal thickness of at least 400 micrometres
Source: Eye (Lond). 2025 Sep 19;39(16):2963–72. doi: 10.1038/s41433-025-04015-6 (PMC12583803; doi:10.1038/s41433-025-04015-6)
Supplement: Supplementary file 1 — Supplementary material [file 41433_2025_4015_MOESM1_ESM.pdf]

SUPPLEMENTARY MATERIAL

Figure S1. Forest plot showing the mean difference in BCVA for diabetic macula oedema (CRT≥400μm) in LogMAR.

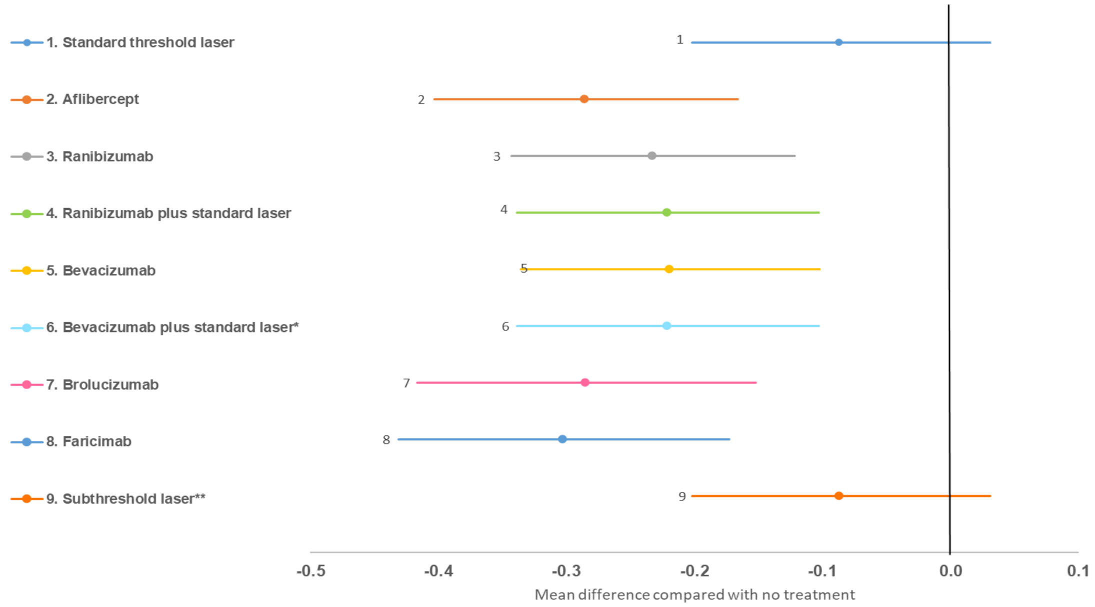

BCVA (best-corrected visual acuity); CRT (central retinal thickness); LogMAR (logarithm of the minimum angle of resolution).
The confidence intervals around the estimates for lasers include zero, indicating uncertainty in the effectiveness of laser compared with no treatment.

Table S1. Treatment effectiveness compared with no treatment.

Treatment	Mean difference at 1 year in LogMAR [95% CI]	Mean annual change in LogMAR	3-monthly prob. of gaining one health state[a]	3-monthly prob. of losing one health state[a]
No treatment	-	-	3.50%	4.50%
Aflibercept	-0.286 [-0.404 to -0.167]	-0.293	7.27%	1.67%
Bevacizumab	-0.220 [-0.336 to -0.103]	-0.227	7.66%	2.42%
Brolucizumab	-0.285 [-0.417 to -0.153]	-0.292	7.28%	1.68%
Faricimab	-0.303 [-0.431 to -0.173]	-0.310	7.11%	1.51%
Ranibizumab[b]	-0.234 [-0.343 to -0.123]	-0.240	7.62%	2.25%
Standard threshold laser[c]	-0.087 [-0.202 to 0.030]	-0.094	7.15%	4.40%
Subthreshold micropulse laser[c]	-0.087 [-0.202 to 0.030]	-0.094	7.15%	4.40%
Bevacizumab plus standard threshold laser[d]	-0.222 [-0.339 to -0.104]	-0.228	7.66%	2.40%
Ranibizumab[b] plus standard threshold laser[d]	-0.222 [-0.339 to -0.104]	-0.228	7.66%	2.40%

[a] *Assumed that the change in best-corrected visual acuity is normally distributed, the mean difference was added to the mean annual change before converting to 3-monthly transition probabilities of moving up or down by one health state.*
[b] *Ranibizumab biosimilar (Ongavia) was assumed to have the same efficacy as ranibizumab (Lucentis).*
[c] *Subthreshold micropulse laser was assumed to have the same efficacy as standard threshold laser.*
[d] *Bevacizumab plus standard threshold laser was assumed to have the same efficacy as ranibizumab plus standard threshold laser.*
LogMAR (logarithm of the minimum angle of resolution).

Table S2. Treatment discontinuation in diabetic macular oedema.

Year	% Remain on treatment	Treatment-specific discontinuation	Source
0 to 1	100%	No	Clinical consensus [1]
1 to 3	Treatment-specific	Yes	See Table S2a below
3 to 5	75%	Yes	Wykoff et al. [2]
>5	50% (base-case)<br>75% (scenario)<br>25% (scenario)	Yes	Scrutiny panel [3]<br>Wykoff et al. [2]<br>Minimum value expected

Table S2a. Treatment-specific discontinuation in diabetic macular oedema.

Resource	Probability of discontinuation (every 3 months)	Source
Aflibercept	2%	NICE TA346 [4]
Bevacizumab	2%	Assumed same as aflibercept
Brolucizumab	3.5%	NICE TA820 [5]
Faricimab	2%	Assumed same as aflibercept
Ranibizumab[a]	1.9%	NICE TA346 [4]
Standard threshold laser	2.52%	NICE TA346 [4]
Subthreshold micropulse laser	2.52%	Assumed same as standard threshold laser
Ranibizumab[a] plus standard threshold laser	1.9%	Assumed same as ranibizumab[a]
Bevacizumab plus standard threshold laser	2%	Assumed same as aflibercept

[a] *Ranibizumab biosimilar (Ongavia) was assumed to be the same as ranibizumab (Lucentis).*

Table S3. Adverse events associated with treatment in diabetic macular oedema.

Adverse events	Aflibercept	Bevacizumab	Brolucizumab	Faricimab	Ranibizumab	Standard laser	Subthreshold laser	Bevacizumab + standard laser	Ranibizumab + standard laser
Retinal detachment	0.08%	0.13%	0.00%	0.02%	0.05%	0.03%	0.03%	0.13%	0.05%
Retinal tear	0.00%	0.00%	0.00%	0.00%	0.00%	0.00%	0.00%	0.00%	0.00%
Vitreous haemorrhage	0.00%	0.00%	0.00%	0.00%	0.00%	0.00%	0.00%	0.00%	0.00%
Increased intraocular pressure	0.00%	0.00%	0.00%	0.00%	0.00%	0.00%	0.00%	0.00%	0.00%
Glaucoma	0.00%	0.00%	0.00%	0.00%	0.00%	0.00%	0.00%	0.00%	0.00%
Endophthalmitis	0.06%	0.07%	0.07%	0.08%	0.14%	0.03%	0.03%	0.07%	0.14%
Cataracts	0.00%	0.00%	0.00%	0.00%	0.00%	0.00%	0.00%	0.00%	0.00%
Ocular pain	0.13%	0.13%	0.69%	0.12%	0.03%	0.06%	0.06%	0.13%	0.03%
Stroke	0.00%	0.00%	0.00%	0.00%	0.00%	0.00%	0.00%	0.00%	0.00%
Cardiovascular death	0.00%	0.00%	0.00%	0.00%	0.00%	0.00%	0.00%	0.00%	0.00%
**Source**	Virgilli et al. [6]	Virgilli et al. [6]	Virgilli et al. [6]	Virgilli et al. [6]	Virgilli et al. [6]	Virgilli et al. [6]	Assumed same as standard laser	Assumed same as bevacizumab	Assumed same as ranibizumab

Table S4. Cost of adverse events.

Resource	Cost	Probabilistic analysis[a]		Source/Notes
		Distribution	Parameters	
Retinal detachment	£2,314.220	Gamma	μ=96.036<br>σ=24.097	AMD NG82 [7] assumed that 75% of patients would require urgent vitrectomy (weighted average of non-elective long and short stay procedures, BZ84A-B major vitreous retinal procedures, 19 years and over, with CC score 0-2+) and 25% of patients have elective surgery (weighted average of day case procedures, BZ84A-B major vitreous retinal procedures, 19 years and over, with CC score 0-2+).
Retinal tear	£185.608	Gamma	μ=96.036<br>σ=1.933	BZ84A-B major vitreous retinal procedures. Total HRGs, weighted average of CC scores.
Vitreous haemorrhage	£482.840	Gamma	μ=96.036<br>σ=5.028	BZ86B intermediate vitreous retinal procedures, 19 years and over, with CC score 0-1, weighted average of non-elective long and short stay based on TA824 [8].
Increased intraocular pressure	£1,012.241	Gamma	μ=96.036<br>σ=10.540	BZ24D-G non-surgical ophthalmology. Total HRGs, weighted average of CC scores, with and without interventions.
Glaucoma	£883.000	Gamma	μ=96.036<br>σ=9.194	Trabeculectomy BZ17B, service code 130 (ophthalmology), major glaucoma procedures, with CC score 0 (day case) based on the evidence review group discussion for NICE TA349 [9], trabeculectomy is the main procedure used.
Endophthalmitis	£1,520.972	Gamma	μ=96.036<br>σ=15.837	Calculated using the distributions from the AMD NG82 [7] and cataract NG77 [10] guidelines. It was assumed that 18.31% of patients require vitrectomy, 38.46% require urgent vitrectomies, and 17.95% of patients require at least 1 revision and 5.13% require 2 revisions, as reported by Kamalarajah et al. [11]. All patients were assumed to require a vitreous tap (weighted average of procedures BZ87A) based on AMD NG82 [7] committee guidance. Elective vitrectomy was assumed to be the weighted average of elective and day case procedures for BZ84A-B major vitreous retinal procedures, while urgent vitrectomy was assumed to be the weighted average of nonelective long-stay procedures BZ84A-B. It was assumed that 5.5 outpatient visits would be needed based on AMD NG82 [7] consultant led non-admitted face-to-face attendance. Code 130 (ophthalmology). It was also assumed that all patients would require medication of Amikacin 500mg/2ml.
Cataracts	£1,945.466	Gamma	μ=96.036<br>σ=20.258	AMD NG82 [7]) and cataract NG77 [10] guidelines, weighted average of non-elective short stay and day case codes for phacoemulsification cataract extraction and lens implant with CC score 4+, 2-3, 0-1, BZ34A-C.
Ocular pain	£1,012.241	Gamma	μ=96.036<br>σ=10.540	BZ24D-G non-surgical ophthalmology. Total HRGs, weighted average of CC scores, with and without interventions.
Stroke	£3,655.557	Gamma	μ=96.036<br>σ=38.064	AA35A-F, stroke. Total HRGs, weighted average of CC scores.
Cardiovascular death	£598.624	Gamma	μ=96.036<br>σ=6.233	VB99Z, emergency medicine, patient dead on arrival.
Myocardial infarction	£1,596.387	Gamma	μ=96.036<br>σ=16.623	EB10A-E actual or suspected myocardial infarction. Total HRGs, weighted average of CC scores.

[a] *Varied by ±20% when relevant data were not available. AMD (age-related macular degeneration); HRGs (Healthcare Resource Groups).*

Table S5. Number of monitoring visits in diabetic macular oedema.

Treatment	Year 1	Year 2	Year 3	Year 4	Year 5 onwards	Source
No treatment	4.00	4.00	4.00	2.00	2.00	NICE TA349 [9]; NICE TA824 [8]
Aflibercept	10.17	7.63	4.52	4.00	3.20	Average across sources (Table S11)
Bevacizumab	10.89	7.81	4.94	4.00	3.20	Assumed same as ranibizumab
Brolucizumab	6.91	4.11	4.00	4.00	2.00	NICE TA820 [5]
Ranibizumab[a]	10.89	7.81	4.94	4.00	3.20	Average across sources (Table S11)
Faricimab	7.58	4.35	4.00	4.00	4.00	Average across sources (Table S11)
Bevacizumab plus standard laser	12.00	8.00	4.00	4.00	4.00	Assumed same as ranibizumab plus standard laser
Ranibizumab[a] plus standard laser	12.00	8.00	4.00	4.00	4.00	NICE TA274 [12]
Standard threshold laser	4.00	3.33	2.80	2.60	2.45	Average across sources (Table S11)
Subthreshold micropulse laser	4.00	3.00	2.80	2.60	2.45	Lois et al. [13]

[a] *Ranibizumab biosimilar (Ongavia) was assumed to be the same as ranibizumab (Lucentis).*

Table S6. Number of anti-VEGF injections in diabetic macular oedema.

Treatment	Year 1	Year 2	Year 3	Year 4	Year 5 onwards	Source
No treatment	0.00	0.00	0.00	0.00	0.00	Not applicable
Aflibercept	8.16	4.34	2.88	2.13	2.06	Average across sources (Table S12)
Bevacizumab	8.28	4.48	2.28	1.71	1.63	Assumed same as ranibizumab
Brolucizumab	6.91	4.11	2.30	1.20	1.00	NICE TA820 [5]
Faricimab	7.06	4.24	1.97	1.97	1.97	Average across sources (Table S12)
Ranibizumab[a]	8.28	4.48	2.28	1.71	1.63	Average across sources (Table S12)
Standard threshold laser	0.00	0.00	0.00	0.00	0.00	Not applicable
Subthreshold micropulse laser	0.00	0.00	0.00	0.00	0.00	Not applicable
Bevacizumab plus standard laser	7.00	3.50	2.50	1.71	1.63	Assumed same as ranibizumab plus standard laser
Ranibizumab[a] plus standard laser	7.00	3.50	2.50	1.71	1.63	Haig et al. [14]

[a] *Ranibizumab biosimilar (Ongavia) was assumed to be the same as ranibizumab (Lucentis).*

Table S7. Number of laser treatments in diabetic macular oedema.

Treatment	Year 1	Year 2	Year 3	Year 4	Year 5 onwards	Source
No treatment	0.00	0.00	0.000	0.000	0.000	Not applicable
Aflibercept	0.00	0.00	0.000	0.000	0.000	Not applicable
Bevacizumab	0.00	0.00	0.000	0.000	0.000	Not applicable
Brolucizumab	0.00	0.00	0.000	0.000	0.000	Not applicable
Faricimab	0.00	0.00	0.000	0.000	0.000	Not applicable
Ranibizumab[a]	0.00	0.00	0.000	0.000	0.000	Not applicable
Standard threshold laser	2.01	0.60	0.675	0.200	0.150	Average across Haig et al. [14], NICE TA346 [4], Lois et al. [13]
Subthreshold micropulse laser	1.90	0.50	0.675	0.200	0.150	Lois et al. [13]
Bevacizumab plus standard laser	2.01	0.60	0.675	0.200	0.150	Assumed same as standard threshold laser monotherapy
Ranibizumab[a] plus standard laser	2.01	0.60	0.675	0.200	0.150	Assumed same as standard threshold laser monotherapy

[a] *Ranibizumab biosimilar (Ongavia) was assumed to be the same as ranibizumab (Lucentis).*

Table S8. Distribution of subsequent treatment in diabetic macular oedema.

From/To	No treat-ment	Standard laser	Afliber-cept	Ranibiz-umab[a]	Ranibizu-mab[a] + standard laser	Bevaci-zumab	Bevaci-zumab + standard laser	Farici-mab	Brolu-cizumab	Dexame-thasone	Fluocino-lone	Source
Standard laser	78%	-	14.8%	4.24%	-	0.71%	-	0.71%	0.71%	0.4%	0.4%	Lois et al. [13]
Aflibercept	63%	37%	-	-	-	-	-	-	-	-	-	Wells et al. [15]
Ranibizumab[a]	54%	46%	-	-	-	-	-	-	-	-	-	Wells et al. [15]
Ranibizumab[a] + standard laser	-	-	-	-	-	-	-	-	-	-	-	-
Bevacizumab	44%	56%	-	-	-	-	-	-	-	-	-	Wells et al. [15]
Bevacizumab + standard laser	-	-	-	-	-	-	-	-	-	-	-	-
Brolucizumab	63%	37%	-	-	-	-	-	-	-	-	-	Assumed same as aflibercept
Faricimab	63%	37%	-	-	-	-	-	-	-	-	-	Assumed same as aflibercept
Subthreshold laser	81.9%	-	12.7%	3.6%	-	0.6%	-	0.6%	0.6%	-	-	Lois et al. [13]

[a] *Ranibizumab biosimilar (Ongavia) was assumed to be the same as ranibizumab (Lucentis).*

Table S9. Cost of subsequent treatment by first-line regimen.

First-line regimen	Cost of subsequent treatment
No treatment	£0.00
Aflibercept	£40.45
Bevacizumab	£61.22
Brolucizumab	£40.45
Faricimab	£40.45
Ranibizumab	£50.28
Standard threshold laser	£2,691.08
Subthreshold micropulse laser	£2,246.26
Bevacizumab plus standard laser	£0.00
Ranibizumab plus standard laser	£0.00

Table S10. Utility losses associated with adverse events.

Resource	Utility decrement	Event duration	Source
Retinal detachment	0.270	3 months	NICE NG82 [7]
Retinal tear	0.000	Immediate repair	NICE NG82 [7]
Vitreous haemorrhage	0.020	-	NICE TA346 [4]; Pochopien et al. [16]
Increased intraocular pressure	0.000	-	NICE TA346 [4]; Pochopien et al. [16]
Glaucoma	0.000	-	NICE TA346 [4]; Pochopien et al. [16]
Endophthalmitis	0.300	20% experience a 1-year quality-of-life effect and 80% a1.5 months	NICE NG82 [7]
Cataracts	0.142	1 month	NICE NG82 [7]
Stroke	0.000	-	No information identified
Cardiovascular death	0.000	-	No information identified
Myocardial infarction	0.000	-	No information identified
Injection related anxiety	0.071	1 day	NICE TA613 [17]; Dolan et al. [18]

Table S11. Frequency of monitoring visits in diabetic macular oedema.

Treatment	Frequency of monitoring visits					Source/Notes
	Year 1	Year 2	Year 3	Year 4	Year 5	
No treatment	4.00	4.00	4.00	4.00	4.00	NICE TA349 [9]; NICE TA824 [8]; Clinical consensus was that monitoring would be reduced to 2 visits a year after year 3
Standard threshold laser	4.00	4.00	2.60	2.20	1.90	NICE TA346 [4]; Clinical consensus was that maximum monitoring visits would be 4
	4.00	3.00	3.00	3.00	3.00	Maredza et al. [19]: followed-up every 3 to 4 months
	4.00	3.00	-	-	-	Lois et al. [13]: followed-up every 3 to 4 months
Aflibercept	8.50	5.10	5.10	0.00	0.00	Régnier et al. [20]
	8.00	6.30	4.00	4.00	2.00	NICE TA346 [4]
	12.00	9.40	4.00	4.00	4.00	NICE TA799 [3]: capped to 12 visits based on clinical consensus
	8.50	4.00	4.00	4.00	4.00	NICE TA799 [3]: scrutiny panel
	12.00	12.00	4.00	4.00	2.00	NICE TA820 [5]: for years 1 and 2; NICE TA346 [4]: years 3 onwards; Capped to 12 visits based on clinical consensus
	12.00	9.00	6.00	4.00	4.00	Maredza et al. [19]: followed-up every 1 to 3 months
Ranibizumab[a]	12.00	9.00	7.50	0.00	0.00	Régnier et al. [20]
	7.70	5.10	5.10	0.00	0.00	Régnier et al. [20]
	12.00	6.30	4.00	4.00	2.00	NICE TA346 [4]
	12.00	9.30	4.00	4.00	4.00	NICE TA799 [3]: capped to 12 visits based on clinical consensus
	8.50	4.00	4.00	4.00	4.00	NICE TA799 [3]: scrutiny panel
	12.00	12.00	4.00	4.00	2.00	NICE TA820 [5]: for years 1 and 2; NICE TA346 [4]: years 3 onwards; Capped to 12 visits based on clinical consensus
	12.00	9.00	6.00	4.00	4.00	Maredza et al. [19]: followed-up every 1 to 3 months
Ranibizumab[a] plus standard laser	12.00	8.00	4.00	4.00	4.00	NICE TA274 [12]
Bevacizumab	4.00	4.00	4.00	0.00	2.00	Assumed same as ranibizumab
Bevacizumab plus standard laser	12.00	4.00	4.00	4.00	4.00	Assumed same as ranibizumab plus standard laser
Brolucizumab	6.91	4.11	4.00	4.00	2.00	NICE TA820 [5]: assumed equal to injection freq. years 1 and 2; NICE TA346 [4]: for year 3 onwards
Faricimab	8.40	4.70	4.00	4.00	4.00	NICE TA799 [3]
	6.75	4.00	4.00	4.00	4.00	NICE TA799 [3]: scrutiny panel
Subthreshold micropulse laser	4.00	3.00	2.60	2.20	1.90	Lois et al. [13]: followed-up every 3 to 4 months with a total of 7 visits over 2 years; Assumed same as standard threshold laser after year 2

[a] *Ranibizumab biosimilar (Ongavia) was assumed to be the same as ranibizumab (Lucentis).*

Table S12. Frequency of anti-VEGF treatments in diabetic macular oedema.

Treatment	Frequency of Anti-VEGF treatments					Source/Notes
	Year 1	Year 2	Year 3	Year 4	Year 5	
Standard laser	-	-	-	-	-	Not applicable
Aflibercept	8.50	5.100	5.10	0.00	0.00	Régnier et al. [20]
	8.00	4.000	2.30	1.20	1.00	NICE TA346 [4]
	8.55	4.000	4.00	4.00	4.00	NICE TA346 [4]: evidence review group report
	9.20	5.000	2.37	2.37	2.37	NICE TA799 [3]
	8.00	4.000	2.00	2.00	2.00	NICE TA799 [3]: scrutiny panel
	8.50	4.000	2.00	2.00	2.00	NICE TA799 [3]: scrutiny panel
	7.70	5.600	2.30	1.20	1.00	NICE TA820 [5]
	6.00	3.000	3.00	-	-	Talks et al. [21]
	9.00	-	-	-	-	Diabetic retinopathy clinical research network (2015)
Ranibizumab[a]	7.00	3.90	2.90	0.00	0.00	Haig et al. [14]
	7.93	4.00	2.30	1.20	1.00	NICE TA346 [4]
	9.40	5.40	2.17	2.17	2.17	NICE TA799 [3]
	8.00	4.00	2.00	2.00	2.00	NICE TA799 [3]: scrutiny panel
	8.50	4.00	2.00	2.00	2.00	NICE TA799 [3]: scrutiny panel
	7.70	5.60	2.30	1.20	1.00	NICE TA820 [5]
	9.40	-	-	-	-	Diabetic retinopathy clinical research network (2015)
Ranibizumab[a] plus standard laser	7.00	3.50	2.50	0.00	0.00	Haig et al. [14]
Bevacizumab	-	-	-	-	-	Assumed same as ranibizumab
Bevacizumab plus standard laser	7.00	3.50	2.50	0.00	0.00	Assumed same as ranibizumab plus standard laser
Brolucizumab	6.91	4.11	2.30	1.20	1.00	NICE TA820 [5]: for years 1 and 2; NICE TA346 [4]: for year 3 onwards
Faricimab	8.420	4.73	1.90	1.90	1.90	NICE TA799 [3]: base-case
	6.000	4.00	2.00	2.00	2.00	NICE TA799 [3]: scrutiny panel
	6.750	4.00	2.00	2.00	2.00	NICE TA799 [3]: scrutiny panel
Subthreshold laser	-	-	-	-	-	Not applicable

[a] *Ranibizumab biosimilar (Ongavia) was assumed to be the same as ranibizumab (Lucentis).*

References

1. Luckham K, Tebbs H, Claxton L, Burgess P, Dinah C, Lois N, et al. A Markov model assessing the cost-effectiveness of various anti-vascular endothelial growth factor drugs and panretinal photocoagulation for the treatment of proliferative diabetic retinopathy. Eye. 2025:1-9.
2. Wykoff CC, Ou WC, Khurana RN, Brown DM, Clark WL, Boyer DS. Long-term outcomes with as-needed aflibercept in diabetic macular oedema: 2-year outcomes of the ENDURANCE extension study. British Journal of Ophthalmology. 2018;102(5):631-6.
3. National Institute for Health and Care Excellence (NICE). Faricimab for treating diabetic macular oedema. Technology appraisal guidance TA799. 2022 [Available from: https://www.nice.org.uk/guidance/ta799.
4. National Institute for Health and Care Excellence (NICE). Aflibercept for treating diabetic macular oedema. Technology appraisal guidance TA346. 2015 [Available from: https://www.nice.org.uk/guidance/ta346.
5. National Institute for Health and Care Excellence (NICE). Brolucizumab for treating diabetic macular oedema. Technology appraisal guidance TA820. 2022 [Available from: https://www.nice.org.uk/guidance/ta820.
6. Virgili G, Parravano M, Menchini F, Evans JR. Anti-vascular endothelial growth factor for diabetic macular oedema. Cochrane Database of Systematic Reviews. 2014(10).
7. National Institute for Health and Care Excellence (NICE). Age-related macular degeneration. NICE guideline NG82. 2018 [Available from: https://www.nice.org.uk/guidance/ng82.
8. National Institute for Health and Care Excellence (NICE). Dexamethasone intravitreal implant for treating diabetic macular oedema. Technology appraisal guidance TA824. 2022 [Available from: https://www.nice.org.uk/guidance/ta824.
9. National Institute for Health and Care Excellence (NICE). Dexamethasone intravitreal implant for treating diabetic macular oedema. Technology appraisal guidance A349. 2015 [Available from: https://www.nice.org.uk/guidance/ta349.
10. National Institute for Health and Clinical Excellence (NICE). Cataracts in adults: management. NICE guideline NG77. 2017 [Available from: https://www.nice.org.uk/guidance/ng77.
11. Kamalarajah S, Silvestri G, Sharma N, Khan A, Foot B, Ling R, et al. Surveillance of endophthalmitis following cataract surgery in the UK. Eye. 2004;18(6):580-7.
12. National Institute for Health and Care Excellence (NICE). Ranibizumab for treating diabetic macular oedema. Technology appraisal guidance TA274. 2013 [Available from: https://www.nice.org.uk/guidance/ta274.
13. Lois N, Campbell C, Waugh N, Azuara-Blanco A, Maredza M, Mistry H, et al. Standard threshold laser versus subthreshold micropulse laser for adults with diabetic macular oedema: the DIAMONDS non-inferiority RCT. Health Technology Assessment (Winchester, England). 2022;26(50):1.
14. Haig J, Barbeau M, Ferreira A. Cost-effectiveness of ranibizumab in the treatment of visual impairment due to diabetic macular edema. Journal of Medical Economics. 2016;19(7):663-71.
15. Wells J, Columbia SC. A deeper look at protocols S and T. Review of ophthalmology. 2016 [Available from: https://www.reviewofophthalmology.com/article/a-deeper-look-at-protocols-s-and-t.
16. Pochopien M, Beiderbeck A, McEwan P, Zur R, Toumi M, Aballéa S. Cost-effectiveness of fluocinolone acetonide implant (ILUVIEN®) in UK patients with chronic diabetic macular oedema considered insufficiently responsive to available therapies. BMC Health Services Research. 2019;19:1-14.
17. National Institute for Health and Clinical Excellence (NICE). Fluocinolone acetonide intravitreal implant for treating chronic diabetic macular oedema in phakic eyes after an inadequate response to previous therapy. Technology appraisal guidance TA613. 2019 [Available from: https://www.nice.org.uk/guidance/ta613.
18. Dolan P. Modeling valuations for EuroQol health states. Medical care. 1997;35(11):1095-108.
19. Maredza M, Mistry H, Lois N, Aldington S, Waugh N. Surveillance of people with previously successfully treated diabetic macular oedema and proliferative diabetic retinopathy by trained ophthalmic graders: cost analysis from the EMERALD study. British Journal of Ophthalmology. 2022;106(11):1549-54.
20. Régnier SA, Malcolm W, Haig J, Xue W. Cost-effectiveness of ranibizumab versus aflibercept in the treatment of visual impairment due to diabetic macular edema: a UK healthcare perspective. ClinicoEconomics and Outcomes Research. 2015:235-47.
21. Talks S, Stratton I, Peto T, Lotery A, Chakravarthy U, Eleftheriadis H, et al. Aflibercept in clinical practice; visual acuity, injection numbers and adherence to treatment, for diabetic macular oedema in 21 UK hospitals over 3 years. Eye. 2022;36(1):72-7.
